# Racial and ethnic differences in social determinants of health among patients with HCC

**DOI:** 10.1097/HC9.0000000000000735

**Published:** 2025-06-09

**Authors:** Mohammed Al-Hasan, Nicole E. Rich, Gloria Figueroa, Stephanie Marie Garces, Lisa Quirk, Sruthi Yekkaluri, Adam Yopp, Patricia D. Jones, Amit G. Singal

**Affiliations:** 1 Department of Internal Medicine, UT Southwestern Medical Center, Dallas, Texas, USA; 2 Parkland Health, Dallas, Texas, USA; 3 Department of Internal Medicine, Division of Digestive Health and Liver Diseases, U. Miami, Miami, Florida, USA; 4 Sylvester Comprehensive Cancer Center, Miami, Florida, USA; 5 Department of Surgery, UT Southwestern Medical Center, Dallas, Texas, USA

**Keywords:** disparities, health literacy, liver cancer, medical mistrust, treatment barriers

## Abstract

**Background:**

Racial and ethnic minority populations are disproportionately impacted by HCC due to more advanced tumor burden and underuse of treatments. We explored racial and ethnic differences in medical mistrust, barriers to treatment, and health literacy among patients with HCC.

**Methods:**

We conducted a multicenter survey among patients with newly diagnosed HCC between September 2018 and July 2023 at 4 large U.S. health systems. The survey assessed medical mistrust [Group-Based Medical Mistrust Scale (GBMMS)], health literacy (CHEW Assessment of Health Literacy), and barriers to HCC treatment. We performed multivariable logistic regression to evaluate associations between race and ethnicity and survey measures.

**Results:**

Of 1245 eligible patients, 833 (66.9%) completed the survey (45.9% Hispanic, 35.9% White, and 14.2% Black). A higher proportion of Black and Hispanic patients had high medical mistrust than White patients (14.2% and 3.3% vs. 0.7%, respectively; *p*<0.001). In multivariable analysis, Black race (OR: 19.2, 95% CI: 4.2–87.7) but not Hispanic ethnicity (OR: 3.72, 95% CI: 0.80–17.2) was significantly associated with high mistrust. Compared to White patients, Black and Hispanic patients both reported greater barriers to HCC treatment, with the most common barriers being concerns about pain (41.6%), financial burden (37.6%), and time commitment (31.1%). Limited health literacy was reported by 38.1% of patients (46.8% Hispanic, 41.0% Black, 26.2% White; *p*<0.001).

**Conclusions:**

Medical mistrust, barriers to treatment, and limited health literacy are prevalent among Black and Hispanic patients with HCC. Understanding the interplay between race, ethnicity, and these factors is essential to address HCC disparities.

## INTRODUCTION

HCC is the third leading cause of cancer-related death worldwide and a significant cause of mortality among patients with cirrhosis.^1^ HCC prognosis is largely determined by tumor stage at diagnosis. Patients diagnosed at an early stage are eligible for curative treatments and have a median survival rate exceeding 5 years, whereas patients diagnosed at later stages typically survive less than 3 years.^2^

In the United States, HCC disproportionately affects racial and ethnic minorities and individuals with low socioeconomic status. Racial and ethnic disparities in HCC incidence, stage at diagnosis, treatment receipt, and prognosis are well-documented. For example, age-adjusted incidence rates are higher among Hispanic and Black patients (8.9 and 8.2 per 100,000, respectively) compared to White patients (4.3 per 100,000).^3^ Additionally, Hispanic (OR: 0.75, 95% CI: 0.55–1.00) and Black patients (OR: 0.74, 95% CI: 0.56–0.98) are more likely to be diagnosed at later stages.^4^ While HCC surveillance is generally underutilized across all groups, it is particularly low in racial and ethnic minorities and those with low socioeconomic status, contributing to lower early-stage HCC detection in these populations.^5^ Studies also consistently show that racial and ethnic minority groups have lower odds of receiving curative treatments, including liver transplantation and surgical resection, and are more likely to experience treatment delays compared to their White counterparts.^6^,^7^,^8^ Taken together, these factors contribute to a worse prognosis and higher HCC mortality rates in minority populations.^9^

Disparities in health outcomes result from the complex interactions between distal factors (population-level social conditions and policies), intermediate factors (social and physical contexts including social support), and proximal factors (individual demographics and tumor-related biological responses).^10^ While several studies have highlighted racial and ethnic differences in medical mistrust, health literacy, and barriers to care in other cancers,^11^,^12^,^13^,^14^,^15^ these constructs remain under-explored in patients with HCC.^16^ Most studies investigating disparities in HCC have relied on electronic medical records or administrative data, which often fail to capture critical patient-reported information. Herein, we report results from a multicenter survey study that examines patient-reported barriers to HCC care among a racially and ethnically diverse cohort of patients.

## METHODS

### Patient population

As previously described, the Multi-Ethnic Hepatocellular Carcinoma (ME-HCC) cohort includes patients aged 18 years or older newly diagnosed with HCC from 4 large health systems in Texas and Florida: UT Southwestern Medical Center (UTSW), Parkland Health, University of Miami Health System (U. Miami), and Jackson Memorial Hospital.^17^ UTSW and U. Miami are large academic tertiary care referral centers, whereas Parkland and Jackson are safety-net health systems for Dallas and Miami-Dade counties, respectively. HCC diagnosis was confirmed based on characteristic imaging or histology, according to AASLD (American Association for the Study of Liver Diseases) criteria.^18^ Patients with a history of previously treated HCC, overt hepatic encephalopathy precluding survey completion, or primary language other than English or Spanish were excluded. This study was approved by the institutional review boards at UTSW and U. Miami.

### Survey administration

A convenience sample of eligible patients was recruited to complete a survey at a clinic appointment or by telephone between September 2018 and July 2023. A follow-up survey was administered ~1–6 months later, scheduled to follow the patient’s first treatment, if applicable. We used a health disparities conceptual model, adapted from Warnecke et al,^10^ to guide selection of relevant constructs to assess (Supplemental Figure S1, http://links.lww.com/HC9/C15).

Surveys were administered in English and Spanish; validated Spanish versions were used when available, and the remaining surveys were translated.^19^,^20^,^21^,^22^,^23^ The baseline survey contained questions about patients’ demographics (including self-reported race and ethnicity, household income, and education level), and included survey instruments assessing medical mistrust and health literacy. The follow-up survey explored other domains, including health locus of control (ie, a person’s belief about who or what controls their health), patient–doctor communication, and barriers to HCC treatment, including financial toxicity. Each survey took approximately ~30 minutes to complete. Participants received a $10 stipend after completion of the follow-up survey.

Medical mistrust was measured using the Group-Based Medical Mistrust Scale (GBMMS), a 12-item survey scored on a 5-point Likert scale ranging from “strongly disagree” to “strongly agree.” Higher GBMMS scores indicate greater mistrust, with a mean score >3 signifying high mistrust.^11^,^24^ Health literacy was assessed using the CHEW Health Literacy Assessment, which consists of 3 questions scored on a 5-point Likert scale, to assess health literacy in 3 key areas: completing medical forms, reading appointment slips, and relying on a surrogate reader. Higher scores suggest higher health literacy, with scores ≤9 indicating limited health literacy, leading to challenges in understanding and managing their healthcare independently.^20^ Barriers to liver cancer treatment were evaluated using a 7-item survey assessing issues such as denial of the HCC diagnosis, perceived necessity of treatment, doubts about treatment efficacy, time constraints, fear of pain, transportation difficulties, and competing life priorities. Lastly, a 4-item survey adapted from the Powe Fatalism Inventory was used to assess health locus of control,^22^ and a 13-item survey, adapted from the Consumer Assessment of Healthcare Providers and Systems, was used to evaluate patient–doctor communication.^23^

### Data collection

We manually reviewed each patient’s electronic medical record to obtain demographic and clinical data, including liver disease etiology, cirrhosis severity, laboratory data, and tumor burden. Race and ethnicity were self-reported and categorized as non-Hispanic White (White), non-Hispanic Black (Black), Hispanic, and other. Liver disease etiology was classified into mutually exclusive categories and hierarchically categorized as hepatitis C (HCV), hepatitis B (HBV), alcohol-associated liver disease (ALD), other (eg, hemochromatosis), or metabolic dysfunction–associated liver disease (MASLD). The highest level of educational attainment was classified into less than high school, high school diploma or general education development (GED), some college or technical school, bachelor’s degree, or advanced degrees. Employment status was categorized as actively employed, unemployed, retired, or disabled. Ascites and hepatic encephalopathy were classified as none, mild/controlled, or severe/uncontrolled. Laboratory data included platelet count, sodium, creatinine, bilirubin, albumin, and international normalized ratio. Cirrhosis severity was assessed using the Child–Pugh score, and tumors were staged according to the Barcelona Clinic Liver Cancer (BCLC) staging system.^25^

### Statistical analysis

Patient characteristics and survey responses were summarized using descriptive statistics, stratified by race and ethnicity. For survey scales (eg, GBMMS) in which an individual had <25% of responses missing, we used single mean imputation to estimate missing values based on other reported responses for that instrument. Patients with >25% missing data for one or more individual survey scales were excluded from analysis for that scale(s) (n=31 for GBMMS and 8 for health literacy). To compare survey measures across racial and ethnic groups, we used Kruskal–Wallis and chi-square tests for continuous and categorical variables, respectively. We also performed exploratory subgroup analyses stratified by BCLC (early-stage vs. non-early) in the full cohort and by preferred language among Hispanic patients. The primary outcomes of interest were medical mistrust and health literacy, for which we performed univariable and multivariable logistic regression analyses. Multivariable models included variables deemed clinically important a priori, including age, gender, employment, and education. We performed an exploratory analysis to evaluate potential interactions between race and other factors (ie, age, gender, employment, and education) for medical mistrust, using GBMMS as a continuous variable. We then reported stratified analyses for any significant interactions. Statistical significance was defined as *p*<0.05, and all analyses were conducted using SAS version 9.4 (SAS Institute).

## RESULTS

### Patient characteristics

Of 1245 eligible patients, 833 (66.9%) completed the baseline survey. Demographic and clinical characteristics are detailed in Table 1. The median age was 64.2 years, and 631 (75.8%) were men. Survey respondents were racially and ethnically diverse, including 382 (45.9%) Hispanic, 299 (35.9%) White, and 118 (14.1%) Black patients. The most common liver disease etiologies were HCV (41.4%), ALD (24.0%), and MASLD (19.1%); Black patients had higher proportions of HCV, and Hispanic patients had higher proportions of ALD or MASLD than their counterparts (*p*<0.001). More than half (55.3%) of patients had Child–Pugh A cirrhosis, and half (50.9%) had early-stage (BCLC A) HCC. Demographic and clinical characteristics of patients who completed the follow-up survey (n=495; 59.4%) are detailed in Supplemental Table S1, http://links.lww.com/HC9/C16. Follow-up respondents were more likely to have Child–Pugh A cirrhosis (*p*=0.002) and BCLC stage A HCC (*p*<0.001) but otherwise had similar characteristics to those who responded to the baseline survey. Therapies received between baseline and the follow-up survey are recorded in Supplemental Table S2, http://links.lww.com/HC9/C16.

**TABLE 1:** Characteristics of patients with HCC who completed the baseline survey

Characteristic	Frequency (%)	White (n=299) (%)	Hispanic (n=382) (%)	Black (n=118) (%)	Other (n=34) (%)	*p*
Site						<0.001
Jackson	101 (12.1)	20 (6.7)	73 (19.1)	6 (5.1)	2 (5.9)	
Parkland	270 (32.4)	50 (16.7)	140 (36.7)	76 (64.4)	4 (11.8)	
UT Southwestern	285 (34.2)	165 (55.2)	73 (19.1)	22 (18.6)	25 (73.5)	
U. Miami	177 (21.3)	64 (21.4)	96 (25.1)	14 (11.9)	3 (8.8)	
Age, y^a^	64.2 (59.0–69.9)	65 (60.5–71.9)	63.7 (57.6–69.5)	64.2 (60.3–66.8)	65.3 (59.0–70.8)	0.03
Gender						0.13
Women	202 (24.3)	61 (20.4)	111 (29.1)	24 (20.3)	6 (17.7)	
Men	631 (75.8)	238 (79.6)	271 (70.9)	94 (79.7)	28 (82.4)	
Etiology						<0.001
Alcohol-associated	200 (24.0)	64 (21.4)	126 (33.0)	6 (5.1)	4 (11.8)	
HBV	32 (3.8)	6 (2.0)	7 (1.8)	11 (9.3)	8 (23.5)	
HCV	345 (41.4)	138 (46.2)	102 (26.7)	95 (80.5)	10 (29.4)	
MASLD	159 (19.1)	56 (18.7)	95 (24.9)	0 (0.0)	8 (23.5)	
Other/unknown	97 (11.6)	35 (11.7)	52 (13.6)	6 (5.1)	4 (11.8)	
Child–Pugh						0.03
Child A	452 (55.3)	175 (59.7)	185 (49.1)	74 (65.5)	18 (52.9)	
Child B	268 (32.8)	89 (30.4)	138 (36.6)	30 (26.6)	11 (32.4)	
Child C	97 (11.9)	29 (9.9)	54 (14.3)	9 (8.0)	5 (14.7)	
Ascites (present)	331 (39.7)	110 (36.8)	177 (46.3)	32 (27.1)	12 (35.3)	0.001
HE (present)	213 (25.6)	71 (23.8)	118 (30.9)	14 (11.9)	10 (29.4)	<0.001
BCLC stage						0.07
BCLC stage A	424 (50.90)	147 (49.2)	208 (54.5)	51 (43.2)	18 (52.9)	
BCLC stage B	141 (16.9)	57 (19.1)	51 (13.4)	27 (22.9)	6 (17.7)	
BCLC stage C	155 (18.6)	56 (18.7)	64 (16.8)	30 (25.4)	5 (14.7)	
BCLC stage D	113 (13.6)	39 (13.0)	59 (15.5)	10 (8.5)	5 (14.7)	
Education						<0.001
Less than high school	211 (26.0)	34 (11.6)	140 (37.7)	35 (30.2)	2 (6.1)	
High school grad/GED	259 (31.9)	94 (32.2)	102 (27.5)	49 (42.2)	14 (42.4)	
Some college/technical degree	212 (26.1)	98 (33.6)	78 (21.0)	26 (22.4)	10 (30.3)	
Bachelors	83 (10.2)	41 (14.0)	32 (8.6)	3 (2.6)	7 (21.2)	
Advanced	47 (5.8)	25 (8.6)	19 (5.1)	3 (2.6)	0 (0.0)	
Employment						<0.001
Employed	220 (27.1)	94 (32.4)	96 (25.6)	18 (15.8)	12 (36.4)	
Retired	296 (36.5)	129 (44.5)	118 (31.5)	36 (31.6)	13 (39.4)	<0.001
Unemployed	105 (12.9)	13 (4.5)	79 (21.1)	10 (8.8)	3 (9.1)	
Disabled	191 (23.5)	54 (18.6)	82 (21.9)	50 (43.9)	5 (15.2)	
Preferred language						<0.001
English	502 (60.3)	279 (94.2)	73 (19.1)	116 (98.3)	34 (100.0)	
Spanish	263 (31.6)	15 (5.0)	246 (64.6)	2 (1.7)	0 (0.0)	
English and Spanish equally	67 (8.1)	5 (1.7)	62 (16.3)	0 (0.0)	0 (0.0)	<0.001

^a^ Median (25th–75th percentile).

Abbreviations: BCLC, Barcelona Clinic Liver Cancer; GED, general education development; MASLD, metabolic dysfunction–associated steatotic liver disease.

### Medical mistrust

**FIGURE 1:**
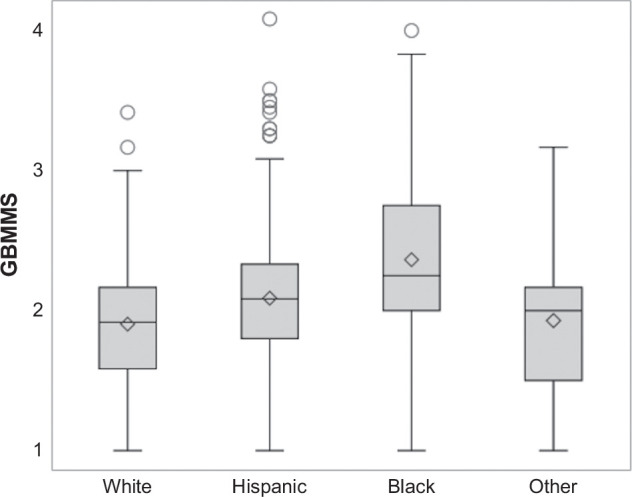
Mean medical mistrust assessed by the GBMMS, stratified by race and ethnicity. Abbreviation: GBMMS, Group-Based Medical Mistrust Scale.

Medical mistrust was reported by 3.9% of patients, which did not significantly differ between patients who only completed baseline surveys compared to those who also completed follow-up surveys (5.2% vs. 2.9%, *p*=0.10) (Supplemental Table S3, http://links.lww.com/HC9/C16). Medical mistrust varied significantly by race and ethnicity, with a greater proportion of Black patients reporting high mistrust (GBMMS >3) compared to Hispanic and White patients (14.2% vs. 3.3% and 0.7%, respectively; *p*<0.001) (Figure 1). Compared to White patients, Hispanic and Black patients were more likely to report that people from their racial/ethnic group are suspicious about modern medicine (3.5% vs. 10.5% and 21.1%, respectively; *p*<0.001), lack trust in healthcare workers (3.1% vs. 8.3% and 12.4%, respectively; *p*<0.001), are suspicious about information from their doctors (4.5% vs. 9.4% and 17.4%, respectively; *p*<0.001), that doctors do not take their complaints seriously (5.9% vs. 10.0% and 20.9%, respectively; *p*<0.001), and that doctors are hiding information from individuals from their racial or ethnic group (5.5% vs. 13.9% and 22.6%, respectively; *p*<0.001). Similarly, Hispanic and Black patients were more likely to report personally being treated poorly or unfairly by doctors because of their race or ethnicity (3.5% vs. 4.8% and 12.2%, respectively; *p*<0.001). In multivariable analyses, Black patients were significantly more likely to report high mistrust (OR: 19.2, 95% CI: 4.2–87.7) compared to White patients, while the association for Hispanic patients was not statistically significant (OR: 3.72, 95% CI: 0.8–17.2) (Table 2). The association between race and ethnicity and GBMMS differed by age (interaction *p*=0.01), with greater differences in mistrust between Black and White patients among older individuals >65 years (mean GBMMS 2.6 vs. 1.8, *p*<0.001) than younger individuals (mean GBMMS 2.3 vs. 1.9, *p*=0.01).

**TABLE 2:** Factors associated with high medical mistrust^a^

Variable	OR (95% CI)
Race (Ref: White)	
Hispanic	3.72 (0.80–17.2)
Black	19.22 (4.21–87.7)
Age (Ref: >65 y)	
≤65 y	1.34 (0.55–3.32)
Gender (Ref: Women)	
Men	0.85 (0.36–2.01)
Education (Ref: Some college or higher)	
High school or less	1.97 (0.77–5.06)
Employment (Ref: Unemployed/disabled)	
Employed	0.73 (0.25–2.13)
Retired	1.21 (0.45–3.23)

^a^ Defined as a Group-Based Medical Mistrust Scale >3.

### Education and health literacy

**FIGURE 2:**
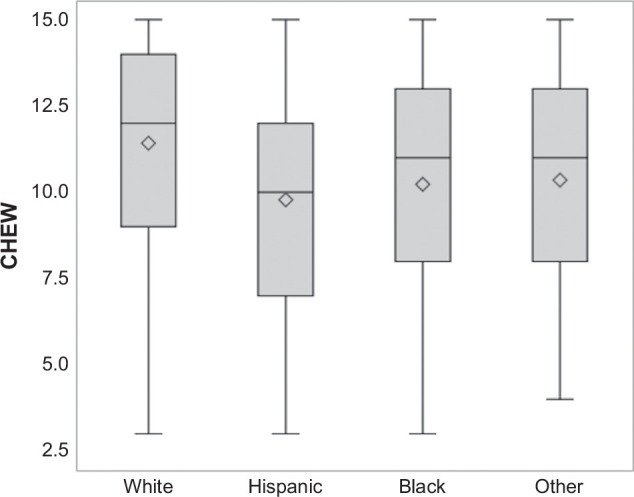
Mean Health literacy, stratified by race and ethnicity. Abbreviation: CHEW, Cultural, Health, and Education Wellness-Health Literacy Assessment.

There were racial and ethnic differences in education level, with 37.7% of Hispanic and 30.2% of Black participants reporting less than high school education, compared to 11.6% of White patients (*p*<0.001). Among Hispanic patients, 71.4% of Spanish-only speakers reported having less than a high school education, compared to 63.4% of English-only speakers and 43.5% of bilingual speakers (*p*<0.001). The mean health literacy score was 10.4 (SD 3.3), with a higher proportion of Hispanic and Black patients having limited health literacy compared to White patients (46.8% and 41.0% vs. 26.2%, respectively; *p*<0.001) (Figure 2). Specifically, a good ability to read was reported by 61.0% of Hispanic and 65.3% of Black patients, compared to 80.2% of White patients (*p*<0.001). Similarly, 49.7% of Hispanic patients reported often needing help to read hospital materials, compared to 35.9% of Black and 29.1% of White patients (*p*<0.001).

Overall, adequate health literacy was observed in 61.9% of patients, although it was higher in patients who completed follow-up surveys than those who only completed baseline surveys (66.7% vs. 54.8%, *p*<0.001) (Supplemental Table S3, http://links.lww.com/HC9/C16). As expected, health literacy also differed by education level (50.6% for high school/GED or less vs. 20.5% for some college or more; *p*<0.001), employment status (49.2% for unemployed/disabled vs. 24.7% for employed patients; *p*<0.001), hospital setting (43.6% for Jackson and 46.6% at Parkland vs. 27.5% at UTSW and 39.2% at U. Miami; *p*<0.001), and preferred language (57.1% of Spanish-speaking vs. 29.9% of English-speaking; *p*<0.001). Among Hispanic patients, a higher proportion of Spanish-only speakers reported limited health literacy compared to English-only and bilingual speakers (58.7% vs. 31.9% and 17.7%; *p*<0.001). In multivariable analysis, Hispanic ethnicity was associated with lower health literacy compared to White patients (OR: 2.0, 95% CI: 1.4–2.9) after adjusting for age, gender, education level, and employment status, whereas the association with Black race was not statistically significant (OR: 1.3, 95% CI: 0.8–2.1) (Supplemental Table S4, http://links.lww.com/HC9/C16). Patients with early-stage HCC were less likely to have limited health literacy than those with larger tumor burden (32.6% vs. 43.7%; *p*=0.001).

### Provider communication

Among the subset who completed the follow-up survey (n=494), most patients reported favorable HCC-related patient–doctor communication, with over 75% of patients reporting their doctor always listened to them carefully (80.6%), always explained things in a way they could understand (79.1%), always encouraged them to ask questions (76.7%), and ensured they understood all the information provided (80.0%). Results were consistent across racial and ethnic groups as well as between those with early-stage HCC compared to those with more advanced stages.

### Barriers to HCC treatment and health locus of control

**FIGURE 3:**
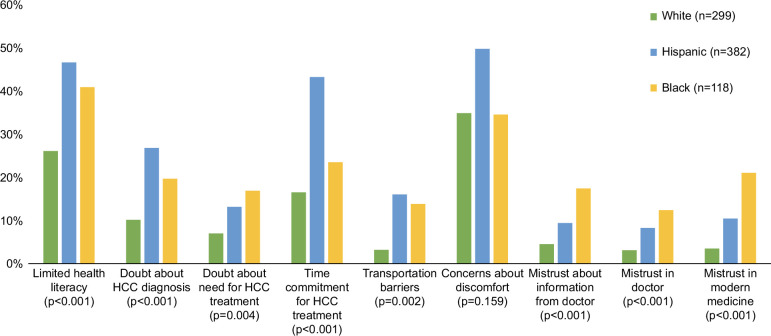
Barriers to HCC-related medical care, stratified by race and ethnicity.

On the follow-up survey, patients reported barriers to HCC treatment (Figure 3), with the most common being concerns about pain (41.6%), required time commitment (31.1%), and uncertainty about their HCC diagnosis (19.5%). Compared to White patients, a higher proportion of Hispanic and Black patients reported concerns about the time commitment (16.6% vs. 43.4% and 23.6%, respectively; *p*<0.001), and uncertainty about the diagnosis (10.1% vs. 26.9% and 19.7%, respectively; *p*<0.001). Over one-third (37.6%) of patients reported financial concerns about paying HCC-related bills, including 10.9% reporting a need to make financial sacrifices and 10.7% accumulating debt due to HCC care, with no significant differences across race/ethnicity. Although difficulty obtaining transportation and doubts about the need for treatment were reported by <15% of patients, these barriers differed by race and ethnicity. Compared to White patients, a higher proportion of Hispanic and Black patients reported transportation difficulty (3.2% vs. 16.1% and 13.9%, respectively; *p*=0.002) and doubts about the need for treatment (7.0% vs. 13.2% and 16.9%, respectively; *p*=0.004).

Among Hispanic patients, a higher proportion of Spanish-only speakers reported concerns about time commitment compared to English-speaking or bilingual individuals (53.0% vs. 20.5% and 32.5%, respectively; *p*=0.001); however, the proportions reporting other barriers did not significantly differ. Barriers including concerns about pain (42.4% vs. 40.4%; *p*=0.24), required time commitment (28.4% vs. 35.0%; *p*=0.24), uncertainty about HCC diagnosis (19.4% vs. 19.7%; *p*=0.17), transportation difficulties (11.4% vs. 10.4%; *p*=0.41), and doubts about need for treatment (12.2% vs. 9.8%; *p*=0.77) did not differ between patients with early-stage HCC and those with more advanced tumor burden.

Many patients expressed fatalistic beliefs, including 44.1% reporting bad events should be accepted without a need to understand why, 74.6% believing people die when it is their time and nothing can change that, and 83.4% feeling that everything that happens to them is part of God’s plan. Health locus of control did not significantly differ between racial and ethnic groups.

## DISCUSSION

To our knowledge, this is one of the largest multicenter studies to examine patient-reported factors that may influence disparities in HCC management, including medical mistrust, health literacy, and barriers to treatment. We observed lower health literacy, greater medical mistrust, and increased barriers to treatment among Hispanic and Black patients. Race and ethnicity were independently associated with several of these constructs after adjusting for differences in age, gender, education, and employment. These data highlight the importance of targeted interventions to address modifiable factors contributing to disparities in HCC treatment receipt and prognosis. Our data are consistent with literature from other cancers,^11^,^12^,^13^,^14^,^26^,^27^ although our study is one of the first to examine these constructs in patients with HCC. Patients with HCC may differ from other cancer types, given that underlying chronic liver disease often necessitates prolonged engagement with hepatology care prior to HCC diagnosis, which offers opportunities to improve health literacy and mitigate medical mistrust. Conversely, the high comorbidity burden, including psychological and substance use conditions, as well as the financial burden experienced by patients with chronic liver disease, could exacerbate barriers to treatment following HCC diagnosis.^28^,^29^

Our findings of greater medical mistrust among Black and Hispanic patients are consistent with extensive prior literature on this topic.^14^,^15^,^30^,^31^ Black and Hispanic patients reported medical mistrust at multiple levels, including modern medicine in general, healthcare institutions, and doctors. However, most patients expressed satisfaction with patient–doctor communication by their HCC team regardless of race or ethnicity, suggesting mistrust may be mitigated once engaged in high-quality multidisciplinary care.^32^ However, our findings that some patients expressed doubt about their HCC diagnosis and need for treatment highlight the need for continued communication and education.

Mistrust likely stems from deeper, historically rooted cultural experiences, as well as individual interactions with doctors and the healthcare system. Higher medical mistrust has been associated with lower cancer screening and treatment delays, which can lead to worse downstream outcomes.^31^ We observed higher levels of mistrust among Black and Hispanic patients, despite patients largely reporting satisfactory communication with doctors. This paradoxical relationship could be related to a selection bias of patients who remained engaged in clinical care and were willing to complete follow-up surveys. Addressing this multifaceted problem may require targeted community-based interventions that engage minority groups and build trust, training programs for doctors in cultural competence, and health systems that foster long-term patient–doctor relationships.

Similarly, lower literacy among Hispanic and Black patients aligns with prior cancer research^33^ and is a major barrier to effective healthcare engagement. Limited health literacy can lead to difficulties understanding cancer-related information or contribute to poor experiences when seeking care.^27^ Low health literacy can also lead to non-adherence with doctors’ recommendations, including treatment or surveillance, which in turn can impact responses and prognosis. Interventions to improve health literacy are critical for reducing disparities in HCC care, whether through community-based programs to reach a broader population or personalized approaches tailored to individual patients during regular clinic visits.^34^

We identified additional barriers to HCC care, including doubts about HCC diagnosis, concerns about treatment-related pain, and time commitment, with Black and Hispanic patients consistently reporting more challenges than White patients. Patient barriers negatively affect outcomes across the cancer care continuum,^35^ including lower cancer screening,^36^ lower treatment adherence,^37^ and poorer overall survival.^38^ Interventions aimed at addressing logistical and financial obstacles to care are a crucial step toward providing equitable care across all populations. Similarly, fatalistic beliefs, which were commonly reported across racial and ethnic groups, can negatively affect cancer outcomes by decreasing engagement in cancer screening^39^,^40^ and contributing to late-stage diagnosis.^41^,^42^ Many participants endorsed believing that God’s plan governs what happens in their life, highlighting an opportunity to develop faith-based interventions or at least consider how one’s faith might impact engagement with healthcare. Interventions aimed at understanding fatalistic beliefs, through culturally sensitive health education and counseling, might empower patients to take a more active role in their care and improve outcomes.

We acknowledge our study’s limitations. First, self-reported survey data are prone to recall bias, nonresponse bias, and social desirability bias, particularly when querying patients about sensitive areas such as medical mistrust and health literacy. Second, recruiting a convenience sample from 4 health systems in the United States may limit the generalizability of our findings, particularly given that the sites were localized to 2 geographical regions (Dallas, Texas and Miami, Florida). Third, as we surveyed only native English and native Spanish speakers, our results may not be generalizable to other populations. However, we believe these limitations are outweighed by the study’s strengths, including our multisite design with a racially, ethnically, and socioeconomically diverse cohort (including various Hispanic ethnicities), high response rate, and unique survey constructs that have not been well described among patients with HCC.

## CONCLUSIONS

There are racial and ethnic differences, with Hispanic and Black patients exhibiting higher medical mistrust and reporting lower health literacy than White patients. These factors, along with logistic and structural barriers to medical care, contribute to reduced engagement with healthcare, which ultimately worsens HCC outcomes for these populations.

## Supplementary Material

## Data Availability

Data supporting the findings of this study are available within the article and its supplementary materials. Other study materials and data related to the study are available from the corresponding author upon reasonable request. This study was conducted with support from NIH MD012565 and R01CA256977. Dr Rich’s research is supported by NIH K08 CA259536. The content is solely the responsibility of the authors and does not necessarily represent the official views of the NIH. The funding agencies had no role in the design and conduct of the study; collection, management, analysis, and interpretation of the data; or preparation of the manuscript. Amit Singal has served as a consultant or on advisory boards for Genentech, AstraZeneca, Eisai, Bayer, Exelixis, Merck, Elevar, Boston Scientific, Sirtex, FujiFilm Medical Sciences, Exact Sciences, HelioGenomics, Roche, Glycotest, Abbott, DELFI, IMCare, and Universal Dx. Adam Yopp has served as a consultant or on advisory boards for Genentech/Roche, AstraZeneca, Merck, France Foundation, Maia, and Intera. Nicole Rich has served as a consultant or on advisory boards for Genentech, AstraZeneca, Eisai, and Exelixis. The remaining authors have no conflicts to report.
