# Supplementary material for: Racial and ethnic differences in social determinants of health among patients with HCC
Source: Hepatol Commun. 2025 Jun 9;9(7):e0735. doi: 10.1097/HC9.0000000000000735 (PMC12150933; doi:10.1097/HC9.0000000000000735)
Supplement: Supplementary file 1 [file hc9-9-e0735-s001.pdf]

Supplemental Figure 1. Conceptual framework of factors contributing to disparate HCC outcomes

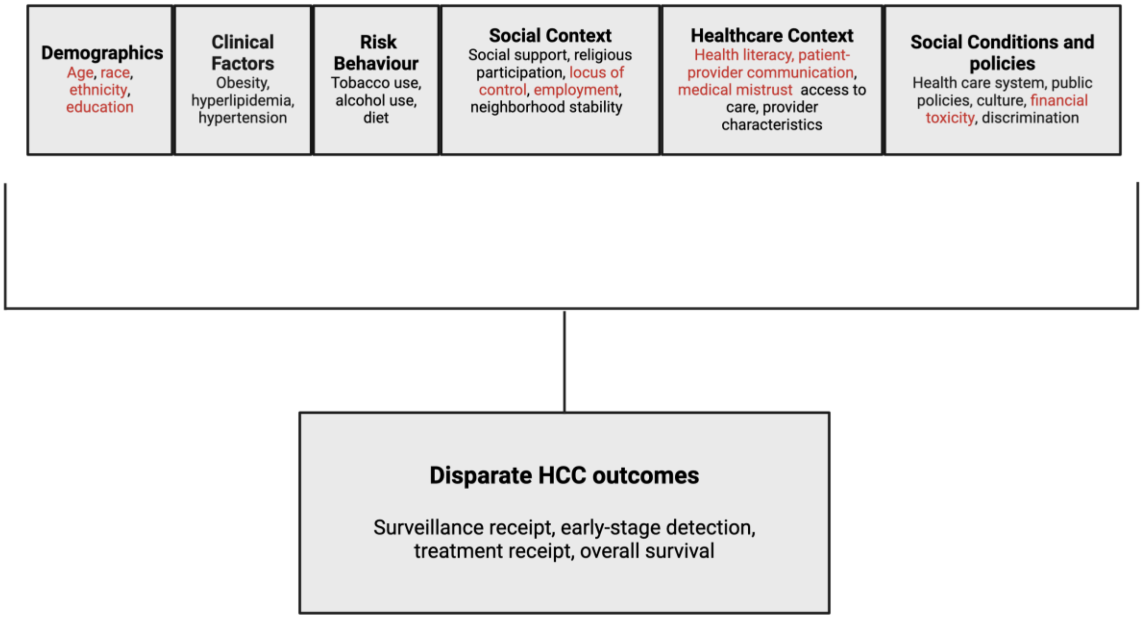

*Measures shown in red were assessed in our study
