# Supplementary material for: Racial and ethnic differences in social determinants of health among patients with HCC
Source: Hepatol Commun. 2025 Jun 9;9(7):e0735. doi: 10.1097/HC9.0000000000000735 (PMC12150933; doi:10.1097/HC9.0000000000000735)
Supplement: Supplementary file 2 [file hc9-9-e0735-s002.docx]

**Supplemental Table 1:** Characteristics of patients who completed the follow-up survey (n=495)

| **Characteristic** | **Frequency**  **(%)** | **White**  **(n=172) (%)** | **Hispanic**  **(n=235) (%)** | **Black**  **(n=74) (%)** | **Other**  **(n=14) (%)** | **p-value** |
| --- | --- | --- | --- | --- | --- | --- |
| **Site** |  |  |  |  |  | <0.001 |
| Jackson | 76 (15.4) | 16 (9.3) | 54 (23.0) | 4 (5.4) | 2 (14.3) |  |
| Parkland | 146 (29.5) | 29 (16.9) | 70 (29.8) | 44 (59.5) | 3 (21.4) |  |
| UT Southwestern | 137 (27.7) | 78 (45.4) | 37 (15.7) | 15 (20.3) | 7 (50.0) |  |
| U Miami | 136 (27.5) | 49 (28.5) | 74 (31.5) | 11 (14.9) | 2 (14.3) |  |
| **Age, years*** | 64.2  (58.8-69.9) | 65.4  (61.4-72.7) | 63.6  (57.2-69.1) | 64.1  (60.3-66.9) | 64.8  (57.4-70.8) | 0.02 |
| **Gender** |  |  |  |  |  | 0.93 |
| Women | 120 (24.3) | 42 (24.4) | 58 (24.8) | 18 (24.3) | 2 (14.3) |  |
| Men | 374 (75.7) | 130 (75.6) | 176 (75.2) | 56 (75.7) | 12 (85.7) |  |
| **Etiology** |  |  |  |  |  | <0.001 |
| Alcohol-related | 118 (23.8) | 36 (20.9) | 77 (32.8) | 3 (4.1) | 2 (14.3) |  |
| Hepatitis B virus | 20 (4.0) | 5 (2.9) | 6 (2.6) | 5 (6.8) | 4 (28.6) |  |
| Hepatitis C virus | 200 (40.4) | 75 (43.6) | 62 (26.4) | 60 (81.1) | 3 (21.4) |  |
| MASLD | 101 (20.4) | 37 (21.5) | 60 (25.5) | 0 (0.0) | 4 (28.6) |  |
| Other/unknown | 56 (11.3) | 19 (11.1) | 30 (12.8) | 6 (8.1) | 1 (7.1) |  |
| **Child Pugh** |  |  |  |  |  | 0.01 |
| Child A | 291 (60.3) | 112 (66.7) | 117 (50.7) | 54 (77.1) | 8 (57.1) |  |
| Child B | 145 (30.0) | 43 (25.6) | 86 (37.2) | 12 (17.1) | 4 (25.6) |  |
| Child C | 47 (9.7) | 13 (7.7) | 28 (12.1) | 4 (5.7) | 2 (14.3) |  |
| **Ascites (Present)** | 176 (35.6) | 56 (32.6) | 101 (43.0) | 15 (20.3) | 4 (28.6) | 0.003 |
| **Hepatic encephalopathy (Present)** | 124 (25.1) | 37 (21.5) | 75 (31.9) | 8 (10.8) | 4 (28.6) | 0.002 |
| **BCLC Stage** |  |  |  |  |  | 0.70 |
| BCLC Stage A | 283 (57.2) | 98 (57.0) | 138 (58.7) | 40 (54.0) | 7 (50.0) | 0.70 |
| BCLC Stage B | 97 (19.6) | 38 (22.1) | 38 (16.2) | 18 (24.3) | 3 (21.4) |  |
| BCLC Stage C | 63 (12.7) | 21 (12.2) | 29 (12.3) | 11 (14.9) | 2 (14.3) |  |
| BCLC Stage D | 52 (10.5) | 15 (8.7) | 30 (12.8) | 5 (6.8) | 2 (14.3) |  |
| **Education** |  |  |  |  |  | <0.001 |
| Less than high school | 111 (22.8) | 15 (8.9) | 72 (31.3) | 23 (31.5) | 1 (7.1) | <0.001 |
| High school grad/GED | 158 (32.5) | 57 (33.7) | 68 (29.6) | 27 (37.0) | 6 (42.9) |  |
| Some college/technical degree | 130 (26.8) | 56 (33.1) | 53 (23.0) | 18 (24.7) | 3 (21.4) |  |
| Bachelors | 55 (11.32) | 24 (14.2) | 24 (10.4) | 3 (4.11) | 4 (28.6) |  |
| Advanced | 32 (6.6) | 17 (10.1) | 13 (5.7) | 2 (2.74) | 0 (0.0) |  |
| **Employment** |  |  |  |  |  | <0.001 |
| Employed | 138 (28.4) | 52 (30.6) | 67 (29.0) | 13 (18.3) | 6 (42.9) |  |
| Retired | 184 (37.9) | 85 (50.0) | 71 (30.7) | 23 (32.4) | 5 (35.7) |  |
| Unemployed | 59 (12.1) | 6 (3.53) | 46 (19.9) | 4 (5.6) | 3 (21.43) |  |
| Disabled | 105 (21.6) | 27 (15.9) | 47 (20.4) | 31 (43.7) | 0 (0.0) |  |
| **Preferred Language** |  |  |  |  |  | <0.001 |
| English | 285 (57.7) | 158 (91.8) | 41 (17.5) | 72 (97.4) | 14 (100.0) | <0.001 |
| Spanish | 162 (32.8) | 11 (6.4) | 149 (63.7) | 2 (2.7) | 0 (0.0) |  |
| English and Spanish equally | 47 (9.5) | 3 (1.7) | 44 (18.8) | 0 (0.0) | 0 (0.0) |  |

*Median (25^th^-75^th^ percentile)

**Abbreviations:** MASLD – metabolic dysfunction-associated steatotic liver disease; BCLC – Barcelona Clinic Liver Cancer; GED – general education development.

**Supplemental Table 2: Types of HCC treatments among patients who completed the follow-up survey (n=495)**

| **Treatment type** | **Frequency (%)** |
| --- | --- |
| Liver Transplantation | 9 (1.8) |
| Surgical resection | 60 (12.1) |
| Local ablation | 63 (12.7) |
| Transarterial radioembolization | 44 (8.9) |
| Transarterial chemoembolization | 99 (20.0) |
| Stereotactic body radiation therapy | 38 (7.7) |
| Systemic therapy | 72 (14.5) |
| Best supportive care | 110 (22.2) |

**Supplemental Table 3: Medical mistrust and health literacy among patients who completed follow-up survey vs. who completed only baseline survey**

| **Variable** | **Overall (%)** | **Baseline surveys only (n=338) (%)** | **Baseline and Follow-up surveys  (n=495) (%)** | **P-value** |
| --- | --- | --- | --- | --- |
| **Health literacy*** |  |  |  | <0.001 |
| Limited health literacy | 314 (38.1) | 150 (45.8) | 164 (33.3) |  |
| Adequate health literacy | 511 (61.9) | 182 (54.8) | 329 (66.7) |  |
| **GBMMS**** |  |  |  | 0.10 |
| Low medical mistrust | 771 (96.1) | 307 (94.8) | 464 (97.1) |  |
| High medical mistrust | 31 (3.9) | 17 (5.2) | 14 (2.9) |  |

*Limited health literacy was defined as CHEW score of ≤ 9.

**High medical mistrust was defined as GBMMS score > 3.

**Supplemental Table 4:** Factors associated with low health literacy*

| **Variable** | **OR** | **95% CI** |
| --- | --- | --- |
| **Race (Ref: White)** |  |  |
| Hispanic | 2.02 | 1.41-2.89 |
| Black | 1.26 | 0.77-2.06 |
| **Age (Ref: > 65 years)** |  |  |
| ≤ 65 years | 0.84 | 0.58-1.22 |
| **Gender (Ref: Women)** |  |  |
| Men | 1.26 | 0.87-1.82 |
| **Education (Ref: Some college or higher)** |  |  |
| High school or less | 3.44 | 2.45-4.83 |
| **Employment (Ref: Unemployed/ disabled)** |  |  |
| Employed | 0.44 | 0.29-0.67 |
| Retired | 0.78 | 0.51-1.18 |

* Defined as CHEW Health Literacy Assessment scale ≤ 9
